# The clinical profile and outcomes of drug resistant tuberculosis in Central Province of Zambia

**DOI:** 10.1186/s12879-024-09238-8

**Published:** 2024-04-01

**Authors:** Evaristo Chanda

**Affiliations:** Department of Public Health, Texila American University, Lusaka, Zambia

**Keywords:** Drug resistant tuberculosis, Clinical Profile, Central province, Zambia

## Abstract

**Background:**

The emergence of Drug Resistant Tuberculosis (DR-TB) is one of the main public health and economic problems facing the world today. DR-TB affects mostly those in economically productive years and prevents them from being part of the workforce needed for economic growth. The aim of this study was to determine the Clinical Profile and Outcomes of DR-TB in Central Province of Zambia.

**Methods:**

This was a retrospective cross sectional study that involved a review of records of patients with confirmed DR-TB who were managed at Kabwe Central Hospital’s Multi-Drug Resistant TB (MDR-TB) Ward from the year 2017 to 2021. 183 patients were managed during this period and all were recruited in the study. Data was collected from DR-TB registers and patient files and then entered in SPSS version 22 where all statistical analyses were performed.

**Results:**

The study revealed that the prevalence of DR-TB among registered TB patients in Central Province was 1.4%. Majority of those affected were adults between the ages of 26 and 45 years (63.9%). The study also found that more than half of the patients were from Kabwe District (60.7%). Other districts with significant number of cases included Kapiri Mposhi 19 (10.4%), Chibombo 12 (6.6%), Chisamba 10 (5.5%), Mumbwa 7 (3.8%) and Mkushi 7 (3.8%). Furthermore, the analysis established that most of the patients had RR-TB (89.6%). 9.3% had MDR-TB, 0.5% had IR-TB and 0.5% had XDR-TB. RR-TB was present in 93.8% of new cases and 88.9% of relapse cases. MDR-TB was present in 6.2% of new cases and 10% of relapse cases. With regard to outcomes of DR-TB, the investigation revealed that 16.9% of the patients had been declared cured, 45.9% had completed treatment, 6% were lost to follow up and 21.3% had died. Risk factors for mortality on multivariate analysis included age 36–45 years (adjusted odds ratio [aOR] 0.253, 95% CI [0.70–0.908] *p* = 0.035) and male gender (aOR 0.261, 95% CI [0.107–0.638] *p* = 0.003).

**Conclusion:**

The research has shown beyond doubt that the burden of DR-TB in Central Province is high. The study recommends putting measures in place that will help improve surveillance, early detection, early initiation of treatment and proper follow up of patients.

## Background

Tuberculosis (TB) is a bacterial disease caused by the bacterium *Mycobacterium tuberculosis* [1]. DR-TB occurs when these bacteria become resistant to the drugs used to treat TB. This means that the drug can no longer kill the TB germ. Factors contributing to the development of resistance include interrupted or inadequate administration of first-line treatment, poor control of infection and easy transmissibility of the drug resistant organism [2, 3]. Other factors include lack of rapid point-of-care diagnostic methods, poor treatment strategies, insufficient second-line drug choices, poor patient adherence to prolonged treatment, stigma, prevalence of Human Immune Virus (HIV), lack of community engagement and poor knowledge of DR-TB among healthcare workers (HCWs) [1–4]. Factors such as individual pharmacokinetics, variable penetration of drugs into the tuberculous lesions and use of standardized regimens in the presence of undiagnosed drug resistance have been suggested by some researchers as the key drivers of DR-TB [5].

DR-TB is spread the same way that drug-susceptible TB is spread [6]. That is the transmission of the germ occurs by airborne spread of infectious droplets and an individual with TB of the lungs coughing, sneezing, talking or singing puts infectious droplets into the air [1]. People nearby may breathe in these bacteria and become infected. Crowding, poor ventilation and poor infection control practices in health facilities and other congregate settings are conducive environments for transmission of TB [6]. Identifying and separating infectious patients, improving ventilation in congregate settings, and initiating effective treatment immediately can help stop transmission of DR-TB [7].

There are different types of DR-TB. The most common types are Rifampicin mono-resistant TB (RMR-TB), Rifampicin-resistant TB (RR-TB), Rifampicin-susceptible, isoniazid-resistant TB (IR-TB), Multi-drug resistant TB (MDR-TB), pre- Extensively drug-resistant TB (preXDR-TB) and Extensively drug-resistant TB (XDR-TB) [8, 9]. RMR-TB is TB resistant to rifampicin and susceptible to isoniazid. RR-TB is TB resistant to rifampicin, regardless of resistance to other drugs. IR-TB is TB resistant to isoniazid and susceptible to rifampicin [10]. MDR-TB is TB resistant to at least both isoniazid and rifampicin. preXDR-TB is TB that is resistant to rifampicin and any fluoroquinolone [11]. XDR-TB is TB that is resistant to rifampicin, plus any fluoroquinolone, plus at least one of the drugs bedaquiline and linezolid [11].

The global burden of DR-TB is not well known. This is due to limited or lack of data in some parts of the world. The World Health Organization (WHO) estimates the burden of DR-TB to have increased between 2020 and 2021, with 450,000 new cases of RR-TB in 2021 [9]. In 2020, it was revealed that 3.3% and 18% of new and previously treated TB cases had MDR-TB respectively and that about half a million people had RR-TB of which 78% developed MDR-TB and 6.2% of MDR-TB patients had XDR-TB [12]. In 2019, about 0.5 million people were diagnosed or notified with MDR-TB/RR-TB. About 50% of MDR/RR-TB cases were reported in India (27%), China (14%) and Russian federation (9%) [12]. Africa, especially the Sub-Saharan Africa is among the regions worst affected by DR-TB. However, data in this area is limited. One study from this region reported an overall resistance to any TB drugs in 18% of the patients and MDR-TB in 9% [13]. Another study underlined that 2.1% of new and 4.6% of previously treated cases were MDR-TB [14]. In the same study, the prevalence of provincial MDR-TB ranged from 1.6% to 5.1% and 4.9% of the reported MDR-TB were XDR-TB [14].

In Zambia, the burden of DR-TB is not well defined because routine surveillance data are scarce despite the country been ranked among the 30 countries with the highest burden of TB and DR-TB in the world [9]. The prevalence of MDR-TB in the country was reported to be 1.8% in 2001 [15]. The proportion of MDR-TB increased from 0.3% among new cases and 8.1% among previously treated cases in 2014 to 2.8% in new cases and 18% in previously treated cases in 2018 [16]. However, data on the regional distribution of DR-TB is limited with no existing data on the burden of DR-TB in the Central Province of Zambia, despite its high TB prevalence and neglected DR-TB status. This study therefore, aimed to assess the clinical profile and outcomes of DR-TB in Central Province of Zambia.

## Methods

### Study design

This was a retrospective cross sectional study. It involved a review of records of patients with confirmed DR-TB who were diagnosed between the year 2017 and 2021 in Central Province of Zambia. 183 patient files were identified and all of them were included in the study. The study period was from 3rd October, 2022 to 30th November, 2022.

### Data collection instruments

A data collection sheet was used to collect data from DR - TB registers and patients’ files at Kabwe Central Hospital’s MDR TB Ward. The collected data included year of diagnosis, age, gender, registration group, site of DR-TB, Type of DR-TB, HIV status and Outcome. The inclusion criteria included confirmed DR- TB cases managed in Central Province and registered at Kabwe Central Hospital’ MDR TB Ward and diagnosed between the year 2017 and 2021. DR-TB was diagnosed using Xpert MTB/RIF. For patients with RR-TB confirmed by Xpert MTB/RIF, samples are sent for First-Line and Second-Line Line Probe Assay (LPA), culture, and phenotypic DST to the reference laboratory. Both Hospital and Community based care is provided to DR-TB patients in Central province. All cases that did not meet the inclusion criteria were excluded from the study.

### Data analysis

After data collection, all uncoded data was coded and then manually entered into Microsoft excel 2016. The data was then extracted into the Statistical Package for Social Sciences (SPSS) version 22.0 where all statistical analysis were performed. Descriptive statistics were computed to determine frequencies of essential parameters. Bivariate analysis and Multivariate logistic regression analysis were also performed to establish the risk factors for the outcome Died among DR-TB patients. Confidence interval was set at 95% and statistical significance was set at a *p*-value of less the 0.05.

### Study variables

#### Dependent variable

The dependent variable in this study was Type of DR-TB. It was measured as a categorical variable.

#### Independent variables

The independent variables were Gender, Age, Registration group, site of DR-TB, HIV status and Disease Outcome. All these were measured as categorical variables.

## Results

### Demographics of participants

- Distribution of Cases of DR-TB based on Gender and Age

The total number of TB cases recorded in Central Province of Zambia from the year 2017 to 2021 was 13,087. Out of this number, 183 cases were DR-TB. Thus the prevalence of DR-TB among registered TB patients in the province was 1.4%. Among the 183 DR-TB cases recruited in the study, 106 (57.9%) were male and 77 (42.1%) were female. Age ranged from 3 months to 84 years with mean age of 35.24 years and standard deviation of 11.83. DR-TB was more prevalent among those who belonged to the age groups 36–45 years (32.2%) and 26–35 years (31.7%). In these two age groups, DR-TB was more prevalent in males than females. This information is summarized in Table 1.

**Table 1:** Distribution of Cases of DR-TB by Age and Gender

			Gender		Total
			F	M	
**Grouped Age**	0–15	Count	3	3	6
		% within Grouped Age	50.0%	50.0%	100.0%
		% within Gender	3.9%	2.8%	3.3%
		% of Total	1.6%	1.6%	3.3%
	16–25	Count	18	11	29
		% within Grouped Age	62.1%	37.9%	100.0%
		% within Gender	23.4%	10.4%	15.8%
		% of Total	9.8%	6.0%	15.8%
	26–35	Count	24	34	58
		% within Grouped Age	41.4%	58.6%	100.0%
		% within Gender	31.2%	32.1%	31.7%
		% of Total	13.1%	18.6%	31.7%
	36–45	Count	25	34	59
		% within Grouped Age	42.4%	57.6%	100.0%
		% within Gender	32.5%	32.1%	32.2%
		% of Total	13.7%	18.6%	32.2%
	Above 45	Count	7	24	31
		% within Grouped Age	22.6%	77.4%	100.0%
		% within Gender	9.1%	22.6%	16.9%
		% of Total	3.8%	13.1%	16.9%
Total		Count	77	106	183
		% within Grouped Age	42.1%	57.9%	100.0%
		% within Gender	100.0%	100.0%	100.0%
		% of Total	42.1%	57.9%	100.0%

Source: Author’s research

(b) Division of Cases of DR-TB by District in Central Province

Most of the cases were from Kabwe District 111 (60.7%). This was followed by Kapiri Mposhi 19 (10.4%), Chibombo 12 (6.6%), Chisamba 10 (5.5%), Mumbwa 7 (3.8%) and Mkushi 7 (3.8%). Other districts had fewer cases as shown in Table 2.

**Table 2:** Distribution of Cases of DR-TB by District in Central Province

District	Frequency (*N* = 183)	Percentage (*N* = 183)
Kabwe	111	60.7%
Kapiri Mposhi	19	10.4%
Mumbwa	7	3.8%
Mkushi	7	3.8%
Chibombo	12	6.6%
Chisamba	10	5.5%
Serenje	4	2.2%
Chitambo	2	1.1%
Other	11	6.0%
Total	183	100.0%

Source: Author’s research

(c) Distribution of Cases of DR-TB by year of diagnosis

**Fig. 1:**
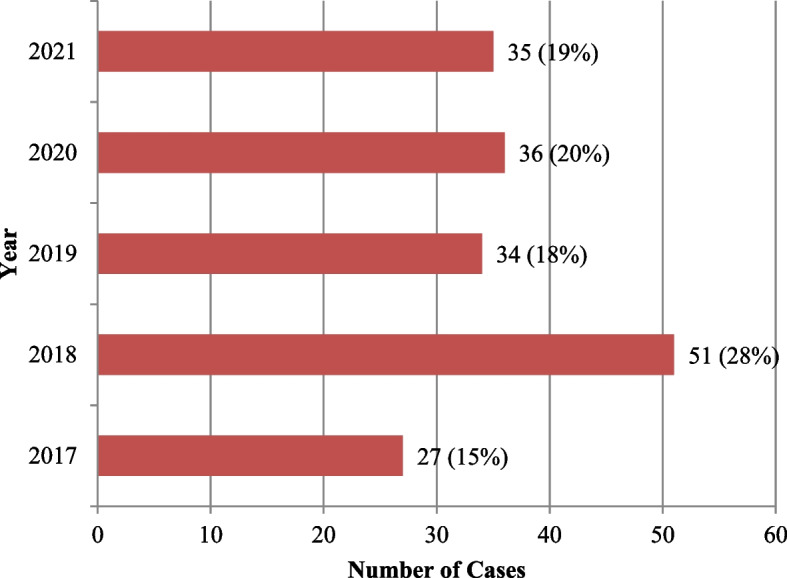
Distribution of Cases of DR-TB by year of diagnosis. Source: Author’s research

Figure 1 shows that the number of patients attended to in 2017 was 27. This number increased to 51 in 2018 and then decreased to 34 in 2019. In 2020 and 2021 the number of cases remained steady at 36 and 35, respectively.

(d) Division of DR-TB Cases based on Registration Group and HIV Status

90 (49.2%) of the patients were Relapse cases while 81 (44.3%) were new cases. 12 (6.5%) belonged to other groups. With regard to the HIV Status of patients, 111 (60.7%) were HIV positive while 61 (33.3%) were HIV negative. The status was unknown in 11 (6.0%) of the patients. Table 3 provides a summary of this information.

**Table 3:** Distribution of Cases by Registration Group and HIV Status

			HIV Status			Total
			Positive	Negative	Unknown	
**Registration Group**	New	Count	50	27	4	81
		% within Registration Group	61.7%	33.3%	4.9%	100.0%
		% within HIV Status	45.0%	44.3%	36.4%	44.3%
		% of Total	27.3%	14.8%	2.2%	44.3%
	Relapse	Count	53	31	6	90
		% within Registration Group	58.9%	34.4%	6.7%	100.0%
		% within HIV Status	47.7%	50.8%	54.5%	49.2%
		% of Total	29.0%	16.9%	3.3%	49.2%
	After loss to follow up	Count	1	1	0	2
		% within Registration Group	50.0%	50.0%	.0%	100.0%
		% within HIV Status	.9%	1.6%	.0%	1.1%
		% of Total	.5%	.5%	.0%	1.1%
	Transfer in	Count	2	0	1	3
		% within Registration Group	66.7%	.0%	33.3%	100.0%
		% within HIV Status	1.8%	.0%	9.1%	1.6%
		% of Total	1.1%	.0%	.5%	1.6%
	Other	Count	5	2	0	7
		% within Registration Group	71.4%	28.6%	.0%	100.0%
		% within HIV Status	4.5%	3.3%	.0%	3.8%
		% of Total	2.7%	1.1%	.0%	3.8%
Total		Count	111	61	11	183
		% within Registration Group	60.7%	33.3%	6.0%	100.0%
		% within HIV Status	100.0%	100.0%	100.0%	100.0%
		% of Total	60.7%	33.3%	6.0%	100.0%

Source: Author’s research

### Types of DR-TB

Most of the patients in this study had RR-TB 164 (89.6%). 17 (9.3%) had MDR-TB, 1 (0.5%) had IR-TB and 1 (0.5%) had XDR-TB. RR-TB was present in 76 (93.8%) of new cases and 5 (6.2%) had MDR-TB. RR-TB was present in 80 (88.9%) of relapse cases and 9 (10%) had MDR-TB. See Table 4.

**Table 4:** Types of DR-TB

			Type of DR TB				Total
			RR TB	IR TB	MDR TB	XDR TB	
**Registration Group**	New	Count	76	0	5	0	81
		% within Registration Group	93.8%	0.0%	6.2%	0.0%	100.0%
		% within Type of DR TB	46.3%	0.0%	29.4%	0.0%	44.3%
		% of Total	41.5%	0.0%	2.7%	0.0%	44.3%
	Relapse	Count	80	1	9	0	90
		% within Registration Group	88.9%	1.1%	10.0%	0.0%	100.0%
		% within Type of DR TB	48.8%	100.0%	52.9%	0.0%	49.2%
		% of Total	43.7%	0.5%	4.9%	0.0%	49.2%
	After loss to follow up	Count	1	0	0	1	2
		% within Registration Group	50.0%	0.0%	0.0%	50.0%	100.0%
		% within Type of DR TB	0.6%	0.0%	0.0%	100.0%	1.1%
		% of Total	0.5%	0.0%	0.0%	0.5%	1.1%
	Transfer in	Count	1	0	2	0	3
		% within Registration Group	33.3%	0.0%	66.7%	0.0%	100.0%
		% within Type of DR TB	0.6%	0.0%	11.8%	0.0%	1.6%
		% of Total	0.5%	0.0%	1.1%	0.0%	1.6%
	Other	Count	6	0	1	0	7
		% within Registration Group	85.7%	0.0%	14.3%	0.0%	100.0%
		% within Type of DR TB	3.7%	0.0%	5.9%	0.0%	3.8%
		% of Total	3.3%	0.0%	0.5%	0.0%	3.8%
Total		Count	164	1	17	1	183
		% within Registration Group	89.6%	0.5%	9.3%	0.5%	100.0%
		% within Type of DR TB	100.0%	100.0%	100.0%	100.0%	100.0%
		% of Total	89.6%	0.5%	9.3%	0.5%	100.0%

Source: Author’s research

### Distribution of outcomes of DR-TB cases

The overall number of patients who had completed treatment at the time the study was done was 45.9%. Yearly distribution was 9 (33.3%), 37 (72.5%), 23 (67.6%) and 15 (41.7%) in 2017, 2018, 2019 and 2020, respectively. Majority of those who completed treatment were male. The study also found that 16.9% of the patients were declared cured overall. The numbers of patients cured in 2017, 2018, 2019 and 2020 were 4 (14.8%), 13 (25.5%), 11 (32.4%) and 3 (8.3%), respectively. This outcome also showed male predominance. For these two outcomes, it should be noted that cases for the year 2021 were not included because patients were still on treatment.

The study further revealed that 6% of the patients overall, defaulted. The patients who defaulted were mostly male and distributed as 3 (11.1%), 4 (7.8%), 1 (2.9%), 0 (0%) and 3 (8.6%) in 2017, 2018, 2019, 2020 and 2021, accordingly. Those who had died were 39 (21.3%). Overall distribution according to year was 14 (51.9%), 9 (17.6%), 8 (23.5%), 3 (8.3%) and 5 (14.3%) in 2017, 2018, 2019, 2020 and 2021, respectively. The study showed that more female than male patients died during the period under consideration. Refer to Table 5.

**Table 5:** Distribution of Treatment Outcomes among Patients

	Outcomes (*N* = 183)								
**Year (*N*** **= 183)**	Cured		Treatment Completed		Lost to follow up		Died		**Total**
	*M*	*F*	*M*	*F*	*M*	*F*	*M*	*F*	
2017	3	1	6	3	3	0	6	8	30
2018	7	6	21	16	4	0	3	6	63
2019	8	3	15	8	1	0	2	6	43
2020	1	2	9	6	0	0	1	2	21
2021	SoT	SoT	SoT	SoT	2	1	4	1	8
**Total**	19	12	51	33	10	1	16	23	**165**

*SoT* Still on Treatment, *M* Male, *F* Female

18 patients were SoT

Source: Author’s research

### Predictors of mortality among DR-TB patients

A logistic regression analysis was conducted to ascertain the effects of age, gender, residential area (District), Registration Group, Type of DR-TB and HIV Status on the outcome Died. Factors were first assessed using bivariate analysis and then entered in a multivariate logistic regression model. Variables with *p*-value ≤0.05 on multivariate regression were considered as statistically significant risk factors for DR-TB mortality. The regression model was statistically significant, χ2 = 44.32, *p* = 0.003. The model explained 33.3% (Nagelkerke *R*^*2*^) of the variance in the outcome Died and correctly classified 79.8% of cases.

On bivariate analysis, risk factors for mortality among DR-TB patients were; male gender (odds ratio [OR] 0.417, 95% CI [0.203–0.859] *p* value = 0.018) and Negative HIV Status OR 0.208, 95% CI [0.052–0.827] *p* value = 0.026. See Table 6. On multivariate analysis, risk factors for mortality included age 36–45 years (adjusted odds ratio [aOR] 0.253, 95% CI [0.70–0.908] *p* = 0.035) and male gender (aOR 0.261, 95% CI [0.107–0.638] *p* = 0.003). See Table 6.

**Table 6:** Logistic regression model of the risk factors associated with the mortality among patients with DR-TB

Characteristics	Un adjusted OR (95% CI)	*p*-Value	Adjusted OR (95% CI)	*p*-Value
**Age**				
• 0–15	0 [0.00]	0.999	0 [0.00]	0.999
• 16–25	0.599 [0.171–2.101]	0.424	0.261 [0.058–1.165]	0.078
• 26–35	1.095 [0.407–2.946]	0.857	0.616 [0.188–2.019]	0.423
• 36–45	0.587 [0.205–1.682]	0.321	0.253 [0.70–0.908]	0.035
• Above 45	Reference Category		Reference Category	
**Gender**				
• Male	0.417 [0.203–0.859]	0.018	0.261 [0.107–0.638]	0.003
• Female	Reference Category		Reference Category	
**District**				
• Kabwe	0.535 [0.145–1.973]	0.348	0.544 [0.131–2.252]	0.401
• Kapiri Mposhi	0.808 [0.169–3.858]	0.789	0.842 [0.150–4.710]	0.845
• Chibombo	0.159 [0.015–1.732]	0.131	0.246 [0.019–3.218]	0.285
• Chisamba	0.438 [0.061–3.160]	0.413	0.438 [0.037–5.210]	0.513
• Other	Reference Category		Reference Category	
**Registration Group**				
• New	3.088 [0.00]	0.999	5.151 [0.00]	0.999
• Relapse	5.546 [0.00]	0.999	9.597 [0.00]	0.999
• After LTF	2.610 [0.00]	0.999	1.839 [0.00]	0.999
• Transfer in	8.077 [0.00]	0.999	8.320 [0.00]	0.999
• Other	Reference Category		Reference Category	
**Type of DR-TB**				
• RR – TB	0.00	1	11.232 [0.00]	1.000
• IR – TB	0.00	0.999	24.576 [0.00]	1.000
• MDR – TB	0.00	1	23.437 [0.00]	1.000
• XDR- TB	Reference Category		Reference Category	
**HIV Status**				
• Positive	0.349 [0.098–1.239]	0.103	0.229 [0.047–1.120]	0.069
• Negative	0.208 [0.052–0.827]	0.026	0.190 [0.034–1.070]	0.060
• Unknown	Reference Category		Reference Category	

*OR* Odds Ratio, *CI* Confidence Interval and *LTF* loss to follow up

There was no missing Data. Total number of cases = 183

Source: Author’s research

## Discussion

### Epidemiology of DR-TB

Drug resistant Tuberculosis is a major public health concern worldwide. The impact of this disease is especially felt in developing countries like Zambia. In this study, the prevalence of DR-TB among registered TB patients in Central Province of Zambia was 1.4%. This prevalence is high when compared to that reported in Luapula and Northern provinces of Zambia of 1% and 1.1%, respectively [17]. However, it is much better than the prevalence reported in other provinces such as Lusaka (43.8%) [17], Copperbelt (46.0%) [12] and Southern province (5.9%) [18]. Monde et al. [19] in their study reported an overall prevalence of DR-TB of 23.5% in Southern and Eastern Provinces of Zambia. As can be seen above, Lusaka and Copperbelt are the two provinces with the highest prevalence of DR-TB. This could be due to the fact that majority of the resources are concentrated in these two regions when compared to other areas. Ngoma [17] also notes that more drug sensitivity tests (DST) are done in these two provinces when compared to other provinces. There is therefore need for equal distribution of resources in all provinces, especially laboratory facilities, machines and reagents to ensure that more tests are conducted and DR-TB cases are not missed. This should be coupled with increased manpower and constant supply of anti-tuberculosis drugs. When compared to studies conducted in other parts of the world, the prevalence of DR-TB for Central province is still low. For example, a study from South Africa reports a provincial DR-TB prevalence of between 1.6% and 5.1% [20]. Another study from Mozambique found an overall resistance to any TB drugs in 18% of the patients [13]. In England, Park et al. [21] reports a DR-TB prevalence of 1.8%. The differences in the prevalence in these studies could be attributed to diversity in diagnostic methods, differences in study methodology, prevalence of HIV and geographical changes [19].

The total number of cases in the study period was 183. The number of cases peaked from 27 in 2017 to 51 in 2018 due to intensified case finding efforts. They then decreased to 34 in 2019 then remained steady at 36 and 35 in 2020 and 2021, respectively. This shift could be due to the Covid-19 pandemic which was characterized by heavy restriction in movement and prolonged quarantine periods leading to decreased efforts in finding cases. The Ministry of Health of Zambia also acknowledges the impact of Covid-19 on TB case finding and state that the TB burden may have increased during the pandemic due to missed cases [22]. Other negative impacts of Covid-19 on TB and DR-TB have also been highlighted in the WHO’s 2022 Global Tuberculosis Report [9].

This investigation also revealed that majority of those affected by DR-TB were between the ages of 26 and 45 years. In this age group, DR-TB was more prevalent in males than females. Similar findings have been reported by several studies [17, 23, 24]. These results are worrying because this age group represents people who are in their most economically productive years and DR-TB limits their ability to participate in the workforce which compromises productivity. This claim is supported by the findings of The Economist Intelligence Unit Limited [25]. The WHO adds that due of the nature of TB, patients and households can face severe indirect and direct financial and economic costs which greatly affect their ability to access diagnosis and treatment, and to complete treatment successfully [10]. One study found that households of patients with DR-TB faced catastrophic costs due to the illness driven by income loss while accessing TB services, nutritional supplements and medical costs [26]. It is therefore important for the government of Zambia and various stakeholders to dedicate more resources to eliminating this deadly but curable disease. This will help meet the third target of the WHO End TB strategy, that no TB patient or their households should face catastrophic total costs as a result of the disease [9, 26]. Wen et al. [27] suggest that material support is feasible and effective to improve treatment success for DR-TB patients combined with other social support interventions.

With regard to the geographical distribution of patients, the study disclosed that more than half of the patients were from Kabwe District (60.7%) making it a hotspot in Central Province. Some districts with significant number of patients with DR-TB included Kapiri Mposhi 19 (10.4%), Chibombo 12 (6.6%), Chisamba 10 (5.5%), Mumbwa 7 (3.8%) and Mkushi 7 (3.8%). Other districts had reported fewer or no patients with DR-TB. These results are important because they reveal a need for increased surveillance, screening and proper management of DR-TB especially in Kabwe which is a transit city. Other districts within the province recorded no or few cases of DR-TB. One explanation for this could be that most of the cases are missed. Majority of the health facilities in these districts lack the resources and expertise required to diagnose and manage DR-TB. Research shows that about 3.6 million people with TB are missed by health systems every year and therefore may not get adequate care they need and that only one in four MDR-TB cases are detected [28]. The Ministry of Heath of Zambia reported that at least 32% of the estimated new and relapse cases of TB were missed in 2020 [22]. Isara and Akpodiete [4] in their study identified poor knowledge of TB among healthcare workers as a contributing factor to the development of DR-TB. Therefore, educational programs for TB and DR-TB should be re-structured. These programs should target healthcare workers in rural areas who have limited access to updated information in as far as the diagnosis and management of DR-TB is concerned. This will promote early detection of DR-TB and improve treatment outcomes among patients.

Last but not last, the study established that about half of the patients managed for DR-TB were relapse cases (49.2%) and 44.3% were new cases. 60.7% of the patients were HIV positive, 33.3% were HIV negative and 6.0% did not know their status. However, the study did not find any correlation between HIV status and the outcome Died. Some of these results are in line with what is in the literature. For example, one study from Mali also found that HIV was not a risk factor for DR-TB [29]. Several other studies found no relationship between HIV status and the outcome of DR- TB as well [30–32]. This finding is contrary to what almost all studies of this nature find which is a positive relationship between HIV-status and DR-TB [33–39]. Most of these studies reveal poor outcomes among DR-TB patients co-infected with HIV, especially those not on treatment or those with poor adherence. The finding in this study could be due to good adherence to HIV treatment among patients co-infected with DR-TB and HIV. However, this claim cannot be relied upon as no data on adherence to DR-TB or HIV treatment was collected in this study. Nonetheless, the impact of HIV on DR-TB has been well documented in the literature and cannot be ignored. Thus, there is need for timely and adequate treatment of DR-TB and HIV infection. The number of DR-TB relapse cases in Central province is also high and should be addressed. Better relapse results have been reported by Mwiinga [40]. Measures need to be put in place to ensure that initial TB is treated effectively and patients followed up adequately. Measures also need to be put in place to ensure proper treatment of DR-TB. These should include early initiation of treatment and prevention of loss to follow up before and after treatment initiation [41].

### Types of DR-TB

Drug – resistant TB is TB resistant to any TB drug [8]. There are different types of DR-TB. These include RMR-TB, MDR-TB, RR-TB, PreXDR-TB and XDR-TB [8, 42]. DR – TB can also be pulmonary or extra pulmonary. Pulmonary DR-TB is confined to the lungs. Extrapulmonary DR-TB is a rare manifestation of disseminated TB and carries a high mortality [43].

The investigation showed that most of the patients had RR-TB (89.6%). 9.3% had MDR-TB, 0.5% had IR-TB and 0.5% had XDR-TB. RR-TB was present in 93.8% of new cases and 88.9% of relapse cases. MDR-TB was present in 6.2% of new cases and 10% of relapse cases. Among all cases, only 1 was classified as being extra-pulmonary DR-TB. The cases of RR-TB are high and cases of MDR-TB are slightly low in Central Province when compared with studies conducted in different parts of the country [12, 18, 40]. The differences in results could be due to heavy reliance on Xpert MTB/RIF in diagnosis DR-TB and delay in receiving results from TB reference laboratories. Despite its advantages, Xpert MTB/RIF has the limitation of being able to detect resistance to rifampicin only [44–47]. In Zambia, for patients with RR-TB confirmed by Xpert MTB/RIF, samples are sent for First-Line and Second-Line Line Probe Assay (LPA), culture, and phenotypic DST. The challenge is there is one National TB Reference Laboratory (Chest Disease Laboratory in Lusaka) and two Regional TB Reference Laboratories (University Teaching Hospital in Lusaka and Tropical Disease Research Centre in Ndola) where culture and first-line phenotypic DST as well as first- and second-line genotypic DST through LPA are performed. District and provincial facilities refer specimens for LPA, culture, and DST to these laboratories and then follow-up of results [48]. The government and other stakeholders should ensure that the capacity of each province to perform laboratory tests is improved. Each province should be able to perform tests like First-Line and Second-Line LPA, culture, and phenotypic DST. This will ensure that severe forms of DR-TB such as pre - XDR and XDR which are more difficult and expensive to manage are not missed. This will also help reduce morbidity and mortality from DR-TB significantly. Effective management of DR-TB will in turn reduce the financial burden on the government as well as patients and their families.

### Common outcomes of DR-TB

Research has revealed several outcomes of DR-TB. These are cured, treatment completed, successful outcomes, treatment failure, lost to follow up and died. Among the 183 patients with DR-TB reviewed during the study period, 16.9% had been declared cured, 45.9% had completed treatment, 6% were lost to follow up and 21.3% had died. These statistics are not impressive and more needs to be done to make improvements. Studies conducted in other parts of the world show better cure and treatment completion rates than those recorded in Central Province [49–53]. Zhang et al. [54] suggests that physicians need to pay close attention to high risk patients so that those with poor responses to treatment are identified early and treatment plan adjusted to improve cure rates.

The number of patients who are lost to follow up is also high in this study. Factors that may have contributed to this include stigma against TB [1], Covid-19 pandemic, poverty and lack of knowledge of DR-TB among patients. Kapata et al. [55] in their study also found that majority DR-TB patients are lost to follow up in Zambia. The authors attributed this to the fact that the reference laboratories from where culture and DST are performed are centralized in the country. A study conducted in South Africa identifies delays and loss to follow-up before treatment of drug-resistant tuberculosis as major factors limiting DR-TB control [41]. Other reasons that have been associated with loss to follow up of DR-TB patients include death after diagnosis, drug side effects, unknown addresses, inability to be contacted, lack of awareness of the seriousness of the diseases, alcoholism, social stigma, lack of transport, long distance to health facility, religious beliefs, negative attitude towards treatment, poverty and lack of family support [55–60]. To tackle this problem, Mishra et al. [58] recommends that health staff, family members and community members must make effort to motivate and support DR-TB patients. Watumo et al. [57] suggests establishing social support platforms to help patients to complete TB treatment, especially the elderly and those who travel long distances. Andargie et al. [61] asserts that strengthening the healthcare system and improving patient education are key in reducing the number of patients who are lost to follow up.

The number of patients that died from DR-TB is also high in Central province. Risk factors for mortality on multivariate analysis were male gender and age group 36 - 45 years. Of the patients that died, 12.6% were female and 8.7% were male (21.3%). Similar findings have been observed by several researchers [62–65]. Mohr-Holland et al. [62] attributes this to challenges with treatment adherence among women. Ravichandran et al. [66] in their study found that females were at more risk than males for adverse events from Anti-Tuberculous drugs. The WHO notes that in some settings, women who become ill with TB are stigmatized, discriminated against or ostracized by their families and communities [67]. They add that cultural and financial barriers can also act as major obstacles for women seeking care, so they may delay accessing care until illness is severe [67]. The number of patients that died from DR-TB might be higher than found in this study because most patients are lost to follow up and some are undiagnosed or misdiagnosed. These patients do not receive the appropriate care they require, and thus end up dying. Mortalities can be reduced through improved surveillance, early diagnosis, early initiation of treatment and proper follow up of patients.

This study has several strengths. First, it was the first of its kind reported from Central province in as far as the burden of DR-TB in the region is concerned. Secondly, the sample size is significant enough to establish the clinical profile and outcomes of DR-TB in Central province. However, potential limitations of the study should be considered while interpreting the findings. One limitation is that study population is composed only of patients who are diagnosed through the healthcare system. Therefore, little is known about patients lacking access to health services. Another limitation is that the data analyzed in the study was restricted to the variables that were available on patient records. Consequently, limited variables were analyzed in this study. Thus, the findings should be interpreted with these limitations in mind.

## Conclusion

TB is among the top 10 causes of death in the world. The emergency of DR-TB has made the control of TB difficult especially is poor countries. The current study has established a high burden of DR-TB in Central Province of Zambia. Majority of those affected are economically active individuals who are between the ages of 26 and 45 years. The common types of DR-TB found in the study are RR-TB and MDR-TB. The investigation has also established a high default and mortality rate among DR-TB patients which may have been made worse by the Covid-19 pandemic. Risk factors for mortality included age 36–45 years and male gender.

## Recommendations

This study has established a high burden of DR-TB in Central Province of Zambia. To reduce this burden the study makes the following recommendations:

- Educational programs coupled with proper counseling should be extended to patients. Patients should be taught the importance of adhering to treatment. Community leaders and church leaders should be involved in monitoring and providing support to these patients, especially those in rural areas were health facilities are not easy to reach.
- Community- or home-based directly observed treatment (DOT) should be implemented as recommended by the World Health Organization. DOT should be provided by trained lay providers or healthcare workers.
- The public should also be sensitized on DR-TB through educational campaigns on television, radio, social media etc. The public should be aware that TB is curable and patients need support throughout the treatment period. This will help reduce stigma and improve patients’ adherence and outcomes.
- The government of Zambia through the Ministry of Health and other relevant stakeholder should ensure that drugs and diagnostic tools (especially laboratory) are always available. This will help prevent treatment interruptions and missing cases.

## Data Availability

The datasets used and/or analyzed during the current study are not publicly available for legal and ethical reasons. The datasets are available from the corresponding author on reasonable request and only in compliance with the legal provisions. More specifically, approval is needed from the National Health Research Authority of Zambia and Ethics review committee.

## Abbreviations

Covid-19: Coronavirus disease 2019
DR-TB: Drug-resistant tuberculosis
IR-TB: Rifampicin-susceptible, isoniazid-resistant
MDR-TB: Multi-drug resistant tuberculosis
MTB: *Mycobacterium Tuberculosis*
PreXDR-TB: Pre - extensively drug-resistant tuberculosis
RMR-TB: Rifampicin mono-resistant tuberculosis
RR-TB: Rifampicin-resistant tuberculosis
TB: Tuberculosis
WHO: World health organization
XDR-TB: Extensively drug-resistant tuberculosis
